# Impact of Magnesium Salt on the Mechanical and Thermal Properties of Poly(vinyl alcohol)

**DOI:** 10.3390/polym13213760

**Published:** 2021-10-30

**Authors:** Riza Asmaa Saari, Muhammad Shahrulnizam Nasri, Takumitsu Kida, Masayuki Yamaguchi

**Affiliations:** Japan Advanced Institute of Science and Technology, School of Materials Science, 1-1 Asahidai, Nomi 9231292, Japan; rizaasmaa@jaist.ac.jp (R.A.S.); s1910429@jaist.ac.jp (M.S.N.); tkida@jaist.ac.jp (T.K.)

**Keywords:** mechanical properties (B), dynamic mechanical thermal analysis (D), polymers (A), stress/strain curves (B), rheology (D)

## Abstract

The effects of magnesium salts with various anion species on the structure and properties of a poly(vinyl alcohol) (PVA) film were studied. The glass transition temperature of the PVA film increased following the addition of a magnesium salt. Furthermore, the salt greatly enhanced the modulus and yield stress and reduced the crystallinity of the film. These effects were attributed to the strong ion–dipole interactions between the magnesium salts and the PVA chains. The strength of interaction, i.e., the reduction of segmental motions, depended on the anion species in the following order: Mg(ClO_4_)_2_, MgBr_2_, MgCl_2_, Mg(CH_3_COO)_2_, and MgSO_4_. The order corresponded to the Hofmeister series, which predicts the ability to break the structure of water.

## 1. Introduction

Poly(vinyl alcohol) (PVA) is an interesting synthetic biodegradable polymer. It has many attractive properties including water solubility, gas barrier properties, and biocompatibility [1,2,3,4,5,6]. PVA also has some excellent mechanical properties, including a high modulus and a high yield stress. Its advantageous mechanical properties mostly arise from its intermolecular hydrogen bonds and the small size of its hydroxyl groups, which allow the PVA chains to pack densely, especially in the glassy region. This is important for a high-modulus fiber. Further improvements to its mechanical properties will facilitate the use of PVA as an alternative to inorganic fibers such as those made from carbon, glass, or metal [7,8,9]. In particular, an enhanced modulus is attractive for film applications. One way to increase the modulus is to enhance interactions with a salt, i.e., to introduce ion–dipole interactions.

The effects of the addition of a salt to PVA have been studied by some researchers [10,11,12]. According to these researchers, a metal salt inhibits or weakens the crystallinity of PVA, leading to poor cohesive strength. This is an interesting result from the perspective of the development of a melt-processable PVA composition. However, an improvement to the mechanical properties is not expected. Several advanced studies on the effect of salts on polar polymers have been reported recently. Such polymers include ethylene–vinyl alcohol [13], bisphenol-A polycarbonate [14], poly(lactic acid) [15], polyamide 6 [16,17], poly(methyl methacrylate) [18,19,20,21], and a copolymer of vinyl butyral and vinyl alcohol [21]. Studies have revealed that the mechanical properties of these polymers are improved by the addition of a salt. Sako et al. discovered that lithium perchlorate (LiClO_4_) enhanced the modulus, yield stress, and fracture energy of bisphenol-A polycarbonate in the glassy state [14]. Tomie et al. [15] studied the effect of lithium trifluoromethanesulfonate (LiCF_3_SO_3_) on the thermal and mechanical properties of poly(lactic acid). They found that LiCF_3_SO_3_ reduced the segmental motion of polymer chains by ion–dipole interactions, which resulted in a high glass transition temperature (*T_g_*) and a high yield stress [15]. Sato et al. found that the tensile storage modulus of polyamide 6 in the glassy state greatly increased with increasing lithium bromide (LiBr) content, which greatly enhanced the *T_g_* [16]. A similar result was obtained for a copolymer of vinyl butyral and vinyl alcohol, the chain of which contains hydroxyl groups [21]. Such interactions with salts are expected for PVA because its chain contains numerous hydroxyl groups.

In the present study, therefore, we studied the ion–dipole interaction between PVA and various salts. Although lithium salts have been investigated greatly as mentioned, we focused on magnesium salts for the following reasons: (1) Magnesium salts have the smallest cations and the largest lattice energies; they form the strongest and most stable ionic bonds. This contributes to the formation of strong ion–dipole interactions with PVA chains [13]. In general, the main factor that influences the lattice energy of ionic crystals is ionic charge. Furthermore, the lattice energy increases as the charge increases. (2) Most magnesium salts are more cost-effective than other salts such as those of lithium. Therefore, we can expect the emergence of an industrial application more than we can from use of a lithium salt However, to the best of our knowledge, no researchers have focused on the effects of the anion species in magnesium salts on the mechanical properties of PVA films.

Kubo et al. revealed that Mg(NO_3_)_2_ decreased the crystallization rate of PVA. As a result, the film had low crystallinity and, consequently, a low *T_g_*. With the enhancement of segmental motion, therefore, the film behaved like a rubber [10]. This research indicated that Mg(NO_3_)_2_ acts as an effective plasticizer for PVA. However, the effects of the anion species of a salt were not investigated in that study.

Recently, Saari et al. found that the crystallization rate of PVA was greatly decreased by specific salts, i.e., lithium iodide (LiI), lithium bromide (LiBr), lithium chloride (LiCl), and lithium nitrate (LiNO_3_) [4,6,22]. These studies revealed that the Hofmeister series, which is often used to explain the role of ion species in polymer solutions (i.e., water-structure breakers or water-structure markers), could be used to explain the effect of the salt on the hydrogen bonds between PVA chains, as well as the rheological properties of films and aqueous solutions [23,24,25,26,27].

In the present study, we added various magnesium salts to PVA to investigate the rheological properties of its aqueous solution and the crystallization behavior and mechanical properties of its solid state films. We found that the tensile modulus of the films was greatly enhanced by an appropriate amount of a magnesium salt, and the *T_g_* increased. Although PVA films have high moduli, this is only true below the *T_g_*. Therefore, enhancing the *T_g_* of PVA would greatly increase its usefulness. Moreover, the results obtained in the present study differed from those obtained by previous researchers, and the modification of the mechanical properties and *T_g_* of PVA films described herein will increase their usefulness.

## 2. Materials and Methods

### 2.1. Materials

The polymeric material used in the present study was a commercially available PVA, kindly provided by Kuraray Co., Ltd., Japan. Its degree of polymerization was 1700 and its degree of saponification was 99.8 mol %. Magnesium perchlorate (Mg(ClO_4_)_2_) was purchased from Kanto Chemical Co., Ltd., Japan. Magnesium bromide (MgBr_2_) and magnesium acetate (Mg(CH_3_COO)_2_) were purchased from Wako Pure Chemical Industry Ltd., Japan. Magnesium chloride (MgCl_2_) was purchased from Nacalai Tesque, Inc., Japan, and magnesium sulphate (MgSO_4_) was purchased from Tokyo Chemical Industry Co., Ltd., Japan. All salts were used without further purification. Deionized water was used throughout the study.

### 2.2. Sample Preparation

For the aqueous solutions, a magnesium salt was added to PVA solutions at molar ratios of 0, 0.006, 0.012, 0.025, and 0.050, relative to the quantity of PVA hydroxyl groups, and the PVA concentration in water was fixed at 15 wt %. Each aqueous solution was prepared by dissolving 7.5 g of PVA in 42.5 mL of deionized water at 90 °C using a magnetic stirrer operating at 400 rpm. Subsequently, the salt was added, and the solution was stirred continuously at 400 rpm for approximately 3 h until the salt had completely dissolved. To prepare the films, the solutions were cast onto a polytetrafluoroethylene-coated a petri dish with a diameter of 75 mm and preheated at 70 °C for 6 h. The obtained films were dried at 100 °C under vacuum for a further 5 h. Finally, the films were dried again at 80 °C under vacuum for 4 h. The properties of the films were determined immediately after the final drying process to avoid the effects of moisture, which greatly affects the *T*_g_ [28].

### 2.3. Measurements

The rheological properties of the solutions were evaluated using a parallel-plate rheometer with a 40 mm diameter (AR2000ex; TA Instruments, Inc., New Castle, DE, USA). The frequency sweep tests were carried out at 25 °C and 60 °C. The gap between the plates was 1 mm. The plates were covered with a solvent trap system that included a wet filter paper inside the cover, and the top of the upper plate was filled with water to increase the humidity in the system. The sample edges were coated with di-2-ethylhexyl phthalate to inhibit water vaporization [6]. The details of the measurements and the reproducibility were reported in our preceding paper [6].

The film morphology was investigated using a scanning electron microscope (SEM; TM3030Plus; Hitachi, Ltd., Tokyo, Japan). Prior to investigation, each film was coated with Pt and Pd using a sputter coating machine. Energy-dispersive X-ray analysis (EDX) was performed after the coating process.

The water content of each film (200 μm thick; 10 mm wide; 20 mm long) was measured using a coulometric Karl Fischer titrator (899 Coulometer; Metrohm AG, Herisau, Switzerland) following ISO 760. The measurements were repeated three times for each film to confirm reproducibility.

The temperature dependences of the tensile storage modulus *E′* and the loss modulus *E″* were measured between 20 °C and 180 °C using a dynamic mechanical analyzer (Rheogel-E4000; UBM Co., Ltd., Mukō, Japan) following JIS K 7244-4. The frequency and heating rate were 10 Hz and 2 °C/min, respectively. Samples (5 mm wide and 10 mm long) were cut from the film, which was 200 μm thick.

The tensile test was carried out at 23 °C using a tensile machine (EZ-LX HS; Shimadzu Corp., Kyoto, Japan) at a stretching speed of 7 mm/min. Because the sample film was small (75 mm in diameter), we prepared specific dumbbell-shaped samples having 7 mm in the gauge length and 4 mm in the gauge width, which were cut out from the films. The initial strain rate was 1 min^−1^. The measurements were repeated five times for each film, and average values with standard deviations were calculated.

The thermal properties of the films were evaluated using a differential scanning calorimeter (DSC) (DSC8500; PerkinElmer, Inc., Waltham, MA, USA) following ISO 11357-3. The samples were heated from 25 to 270 °C, i.e., the first scan, at a rate of 10 °C/min under a nitrogen atmosphere. DSC evaluations were carried out on samples (approximately 10 mg) that were encapsulated in a hermetically sealed aluminum pan.

Infrared spectra were collected at 23 °C using a Fourier-transform infrared (FT-IR) spectrometer (Spectrum 100; PerkinElmer, Inc.). The measurements were performed in the attenuated total reflection mode using KRS-5 as a crystal and were obtained at a resolution of 4.0 cm^−1^ by averaging 16 scans.

## 3. Results and Discussion

**Figure 1:**
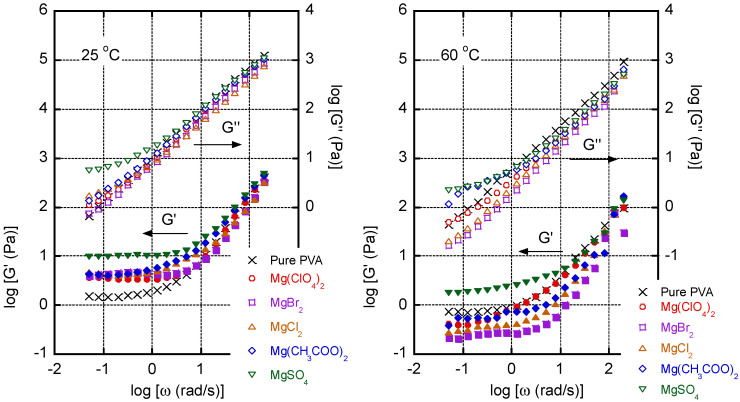
Angular frequency (*ω*) dependence of shear storage modulus *G′* and loss modulus *G″* of aqueous PVA solutions at 25 °C (**left**) and 60 °C (**right**); (x symbols) PVA, (circles) PVA/Mg(ClO_4_)_2_, (squares) PVA/MgBr_2_, (triangles) PVA/MgCl_2_, (diamonds) PVA/Mg(CH_3_COO)_2_, and (inverted triangles) PVA/MgSO_4_. The molar ratio of magnesium salts to PVA hydroxyl groups was 0.05.

Figure 1 summarizes the angular frequency dependence of the oscillatory shear moduli such as storage modulus *G′* and loss modulus *G″* of the solutions with/without a magnesium salt. Each salt was added at a molar ratio of 0.050 relative to the quantity of hydroxyl groups in PVA, which were 20.2 wt % of Mg(ClO_4_)_2_, 17.3 wt % of MgBr_2_, 9.8 wt % of MgCl_2_, 13.9 wt % of Mg(CH_3_COO)_2_, and 12.0 wt % of MgSO_4_.

It was evident that the addition of salt increases the plateau modulus in the *G′* curve at low frequencies and at 25 °C, suggesting that hydrogen bonding between the PVA chains was enhanced by the presence of the salt, as previously demonstrated by Saari et al. in a study on KBr [6]. According to those researchers, K^+^, which, according to the Hofmeister concept, is a water-structure maker, is responsible for the development of a network structure produced by strong hydrogen bonding between the PVA chains [6]. However, at 60 °C, with exception of the MgSO_4_ solution, the plateau moduli were reduced by the addition of salt and the *G″* values decreased. Thus, the addition of magnesium salts to PVA films can either enhance or reduce the plateau modulus depending on the temperature. Therefore, the development of a network structure in aqueous PVA solutions containing magnesium salts is temperature-sensitive. To the best of our knowledge, this has not been reported previously and is pertinent to film preparation and/or wet spinning. In the case of the solution containing MgSO_4,_ which is well-known as a strong water-structure maker, the plateau modulus was enhanced at both temperatures.

**Figure 2:**
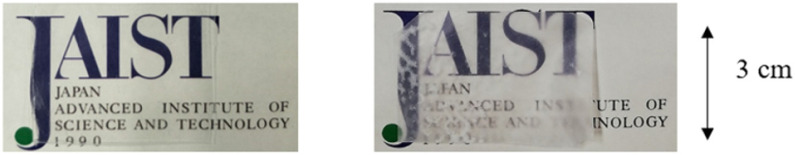
Photographs of the films with 200 μm thickness: PVA/Mg(ClO_4_)_2_ (**left**) and PVA/MgSO_4_ (**right**). Each film had a salt content of 0.05 as a molar ratio relative to the quantity of PVA hydroxyl groups.

The transparencies of films containing various amounts of each salt were evaluated by the naked eye. As shown in the left picture in Figure 2, except for the film containing MgSO_4_, all the films were transparent. In contrast, the films containing MgSO_4_ were opaque, even at a molar ratio of 0.012 (3.2 wt %), owing to light scattering, which became more pronounced as the salt content increased.

**Figure 3:**
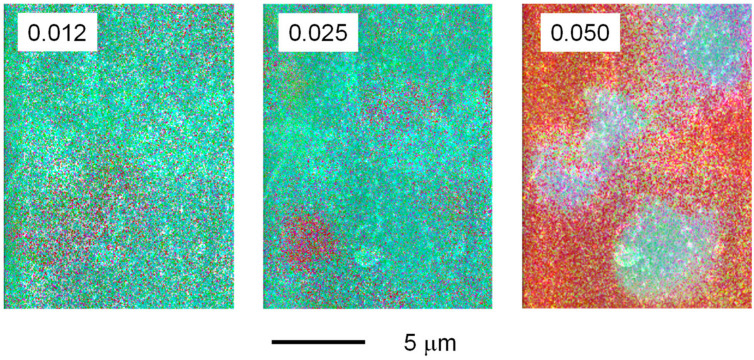
Energy-dispersive X-ray spectroscopy images of the films containing MgSO_4_. The numerals in the figure represent the molar ratios of MgSO_4_.

The distribution of MgSO_4_ in the film was further investigated using EDX. As shown in Figure 3, carbon (blue), oxygen (green), sulfur (red), and magnesium (purple) were clearly detected in the EDX images of the films containing MgSO_4_. Furthermore, it was obvious that salt segregation increased as the MgSO_4_ content increased. Because all the aqueous solutions used to prepare the film samples were transparent, including those containing MgSO_4_, phase separation, i.e., salt segregation, occurred during water evaporation.

The differences in the appearances of the film can be explained by the Hofmeister series; weakly hydrated anions such as ClO_4_^−^ and Br^−^ are categorized as water-structure breakers and are therefore highly soluble in aqueous PVA. In common with the film containing Mg(ClO_4_)_2_, the films containing MgBr_2_ or MgCl_2_ were transparent. In contrast, strongly hydrated anions such as SO_4_^2−^ are categorized as water-structure makers and are poorly soluble in aqueous PVA [20,29]. In the present study, further experiments were not performed using films containing MgSO_4_ because they were not homogeneous. Instead, the properties of films containing Mg(ClO_4_)_2_ were compared with those of films containing Mg(CH_3_COO)_2_, because these two salts are located at different ends of the Hofmeister series, i.e., Mg(ClO_4_)_2_ is a water-structure breaker and Mg(CH_3_COO)_2_ is a water-structure maker.

Generally, water content has a great influence on the mechanical and thermal properties of PVA films. Furthermore, magnesium salts are hygroscopic [30]. Therefore, the effects of salts on the water contents of the films were studied using the Karl Fischer titration method prior to the evaluation of the film properties. Because the measurements were performed after vacuum drying at 80 °C for 4 h, the content of water trapped by the hydroxyl groups in PVA was evaluated [31]. The measurements revealed that the water content of each film was below 1.0 wt % (the experimental error was within 10% for each film). Therefore, the effect of water content on the film properties can be ignored.

**Figure 4:**
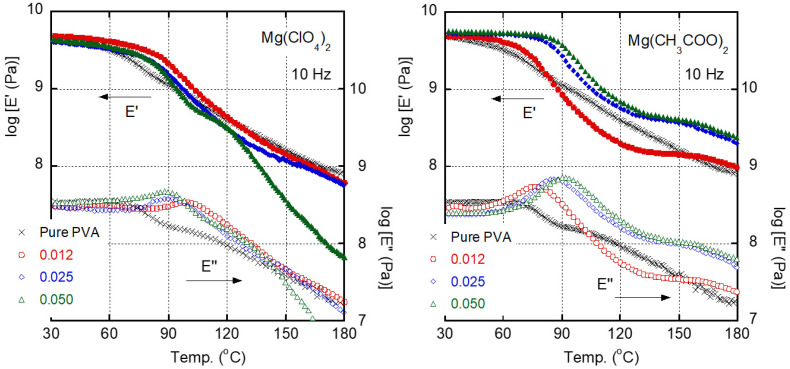
Temperature dependence at 10 Hz of tensile storage modulus *E′* and loss modulus *E″* of films containing various molar ratios of Mg(ClO_4_)_2_ (**left**) or Mg(CH_3_COO)_2_ (**right**); (x symbols) pure PVA, (circles) 0.012 molar ratio, (diamonds) 0.025 molar ratio, and (triangles) 0.050 molar ratio.

The dynamic mechanical properties of the films containing Mg(ClO_4_)_2_ and those containing Mg(CH_3_COO)_2_ are shown in Figure 4. The peak temperature of *E″*, i.e., the *T_g_*, increased markedly. Interestingly, this was most obvious in the film containing a molar ratio of Mg(ClO_4_)_2_ of 0.012 (5.7 wt %). However, the films containing more Mg(ClO_4_)_2_ had slightly lower *T_g_* values than the film with a molar ratio of 0.012. In the case of the films containing Mg(CH_3_COO)_2_, the *T_g_* increased monotonously as the salt content increased. The *T*_g_ shift will be discussed again later.

Figure 4 also demonstrated that the *E′* values are enhanced for most of the films except for the film with a Mg(ClO_4_)_2_ molar ratio of 0.050 (20.2 wt %) and the film with a Mg(CH_3_COO)_2_ molar ratio of 0.012 (3.7 wt %). PVA films have high moduli and are therefore attractive for certain applications. The modulus enhancement is significantly pronounced in the temperature range 60 °C to 100 °C for films containing Mg(CH_3_COO)_2_ molar ratios of 0.025 (7.5 wt %) and 0.050 (13.9 wt %). Among the films containing Mg(ClO_4_)_2_, the modulus enhancement was obvious when the salt content was low, i.e., a molar ratio of 0.012 (5.7 wt %), which corresponds to enhancement of the *T_g_*. For these films, a weak temperature dependence of the *E′* values in the glassy region is responsible for the modulus enhancement in that temperature range. Correspondingly, the glass-to-rubber transition occurred in a narrow temperature range, resulting in a sharp peak in the *E″* curve.

**Figure 5:**
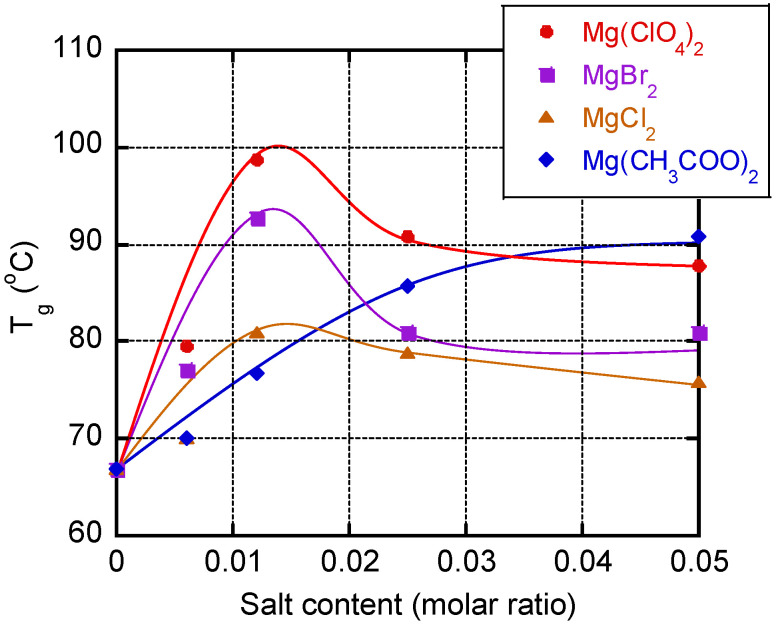
Relationship between the glass transition temperature (*T_g_*) and the salt content.

Figure 5 illustrates the *T_g_* values, i.e., the peak temperatures in the *E″* curve, of the films containing various magnesium salts. It is obvious that the addition of Mg(ClO_4_)_2_, MgBr_2_, or MgCl_2_ greatly enhanced the *T_g_* when the salt content was low, e.g., a molar ratio of 0.012 (5.7 wt % of Mg(ClO_4_)_2_, 4.8 wt % of MgBr_2_, and 2.5 wt % of MgCl_2_), but further addition reduced the *T_g_*.

The results indicate that two main factors determine the *T_g_*. One is the ion–dipole interaction between PVA and the salt, which enhances the *T_g_* by reducing the segmental motion of the PVA chains. The other is the reduction of intermolecular hydrogen bonding between the PVA chains, which promotes segmental motion and reduces the *T_g_*. The latter interaction also reduced the crystallinity of the PVA, as discussed later. In contrast, there was a monotonous increase in *T_g_* in the films containing Mg(CH_3_COO)_2_. Because CH_3_COO^−^ is known as a water-structure maker, the intermolecular hydrogen bonding between the PVA chains is barely affected. Therefore, only one mechanism, i.e., ion–dipole interaction, determines the *T_g_* and *E′* values. The difference in the effect of the salt content must also depend on the dissociation state because Mg(ClO_4_)_2_ dissociates more easily than Mg(CH_3_COO)_2_.

Therefore, the anion species is an important factor in the control of the dynamic mechanical properties of a film, including the *T_g_* and *E′*. This can be explained by the Hofmeister series, i.e., ClO_4_^−^, Br^−^, Cl^−^, and CH_3_COO^−^ arranged by order of effectiveness as a water-structure breaker [32].

**Figure 6:**
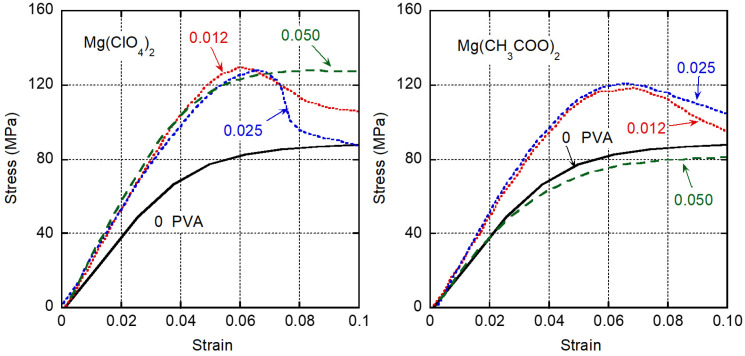
Stress–strain curves of films containing Mg(ClO_4_)_2_ (**left**) and Mg(CH_3_COO)_2_ (**right**) at 23 °C. The numerals in the figure represent the molar ratios of the salts.

The stress–strain curves obtained at 23 °C are shown in Figure 6. Both stress and strain are engineering values. In accordance with the dynamic mechanical properties, the initial tensile moduli of the films containing Mg(ClO_4_)_2_ and those containing Mg(CH_3_COO)_2_ were significantly higher than that of pure PVA (2.44 GPa); Mg(ClO_4_)_2_ had a particularly high initial tensile modulus. Moreover, except for one film the yield stresses of the films containing the salts were much higher than the yield stress of pure PVA. The excellent tensile properties of the films, as summarized in Table 1 with the standard deviation, also make them attractive for practical uses. The film with a Mg(CH_3_COO)_2_ molar ratio of 0.050 (13.9 wt %) had almost the same curve as the pure PVA film, although there was a marked shift in the *T_g_*. Considering that CH_3_COO^−^ is a water-structure maker, salt agglomeration might occur on a small scale, resulting in poor tensile properties.

**Figure 7:**
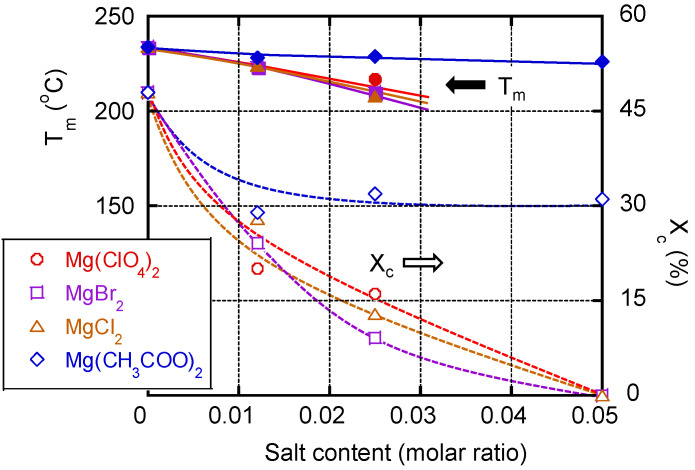
*T_m_* and *X_c_* plotted against the salt content; (circles) PVA/Mg(ClO_4_)_2_, (squares) PVA/MgBr_2_, (triangles) PVA/MgCl_2_, and (diamonds) PVA/Mg(CH_3_COO)_2_.

Figure 7 shows the melting point *T_m_* and the degree of crystallinity *X_c_*—evaluated from the DSC measurements—plotted against the salt content. *X_c_* was calculated using the following equation:

(1) $$X_{c}=\frac{\Delta h_{F}}{\Delta h_{F\text{-}perfect}}\times 100\;(\%)$$

where Δ*h_F-perfect_* is the heat of fusion of a perfect crystal of PVA (152 J/g) [33].

In the case of pure PVA, there was a melting peak at approximately 234 °C with a peak area of 74 J/g (48% crystallinity); these are typical values for a PVA film [34]. The *T_m_* values of the films containing Mg(CH_3_COO)_2_ were 228 °C (molar ratio 0.012), 229 °C (molar ratio 0.025), and 226 °C (molar ratio 0.050), i.e., not significantly different from the *T_m_* of pure PVA.

The degree of crystallization values of the films containing Mg(ClO_4_)_2_ with molar ratios of 0.012 and 0.025 were 20% and 16%, respectively, which were much lower than the degree of crystallization of a pure PVA film (48%). Moreover, a Mg(ClO_4_)_2_ molar ratio of 0.05 provided no/little crystallinity, which was also true for the films containing the same amount of MgBr_2_ or MgCl_2_. Clearly, the addition of a water-structure breaker—e.g., Mg(ClO_4_)_2_, MgBr_2_, or MgCl_2_—reduced the crystallinity.

**Figure 8:**
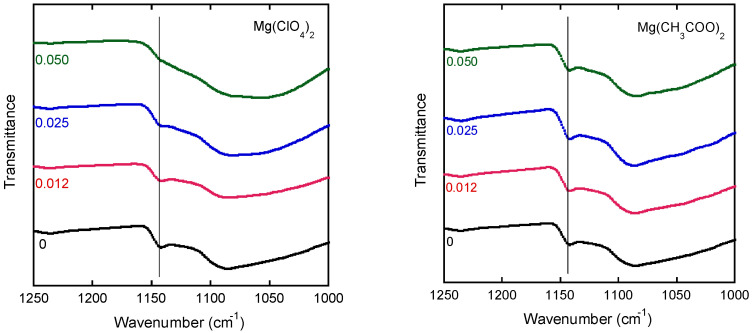
FT-IR spectra of the films containing Mg(ClO_4_)_2_ (left) and Mg(CH_3_COO)_2_ (right) in the wavenumber range 1000–1250 cm^−1^. The numerals in the figure represent the molar ratios of the salts.

The effect of magnesium salts was also studied by FT-IR spectroscopy as shown in Figure 8. The addition of Mg(ClO_4_)_2_ decreased the magnitude of the crystalline band at 1141 cm^−1^. Remarkably, the spectrum did not reveal any crystallinity in the film with a molar ratio of 0.050. In contrast, the intensity of the crystalline band remained strong when the film contained Mg(CH_3_COO)_2_, similar to that of pure PVA. These results corroborate those obtained by DSC.

## 4. Conclusions

The structures and properties of aqueous solutions and solid films of PVA containing magnesium salts were studied. Salts known as water-structure breakers—such as Mg(ClO_4_)_2_, MgBr_2_, and MgCl_2_—promoted ion–dipole interactions at low temperatures, e.g., 25 °C, in the aqueous solution, leading to a high plateau modulus in the low frequency range. However, the interaction becomes weak at high temperatures, e.g., 60 °C. Water-structure makers such as MgSO_4_ also enhanced the plateau modulus at both low and high temperatures because they increased intermolecular hydrogen bonding between the PVA chains. Films containing MgSO_4_ are, however, opaque, owing to light scattering caused by the agglomeration of the salt. The film properties depended heavily on the anion species. The storage modulus was enhanced as the *T_g_* increased in the films containing a small amount of a water-structure breaker salt in which the crystallinity was greatly reduced. In contrast, the modulus enhancement with the *T_g_* shift became obvious as the content of Mg(CH_3_COO)_2_, i.e., a water-structure maker, increased. Tensile testing also revealed an enhancement of the modulus in the presence of the salt, which was accompanied by a sizeable increase in the yield stress. Therefore, the addition of a small amount of a water-structure breaker salt effectively improves the mechanical properties of PVA films—such as rigidity and strength—which must widen their applicability. We will further study the phenomenon to propose a novel material design to increase the modulus and strength of PVA films and fibers using an appropriate salt. Furthermore, the effect of the water absorption should be clarified for the industrial application.

## Figures and Tables

**Table 1:** Tensile properties of the films.

Salt	Molar Ratio (wt %)	Tensile Modulus GPa	Yield Stress MPa
Pure PVA	-	2.4 <0.2>	88 <1>
Mg(ClO_4_)_2_	0.012 (5.7)	3.3 <0.2>	130 <1>
	0.025 (11.3)	3.0 <0.3>	128 <1>
	0.050 (20.2)	3.1 <0.6>	129 <3>
Mg(CH_3_COO)_2_	0.012 (3.7)	2.8 <0.2>	119 <1>
	0.025 (7.5)	2.9 <0.3>	120 <1>
	0.050 (13.9)	2.5 <0.6>	86 <1>

<Standard deviation>.

## Data Availability

Not applicable.
